# Prognostic relevance of induced and spontaneous apoptosis of disseminated tumor cells in primary breast cancer patients

**DOI:** 10.1186/1471-2407-14-394

**Published:** 2014-06-03

**Authors:** Natalia Krawczyk, Andreas Hartkopf, Malgorzata Banys, Franziska Meier-Stiegen, Annette Staebler, Markus Wallwiener, Carmen Röhm, Juergen Hoffmann, Markus Hahn, Tanja Fehm

**Affiliations:** 1 Department of Obstetrics and Gynecology, University of Duesseldorf, Moorenstr. 5, 40225 Duesseldorf, Germany; 2 Department of Obstetrics and Gynecology, University of Tuebingen, Calwerstr. 7, 72076 Tuebingen, Germany; 3 Department of Pathology, University of Tuebingen, Liebermeisterstr. 8, 72076 Tuebingen, Germany; 4 Department of Obstetrics and Gynecology, University of Heidelberg, Voßstr. 9, 69115 Heidelberg, Germany

**Keywords:** Apoptosis, M30, Breast cancer, Survival, Disseminated tumor cell

## Abstract

**Background:**

An imbalance between cell proliferation and programmed cell death can result in tumor growth. Although most systemic cytotoxic agents induce apoptosis in tumor cells, a high apoptotic rate in primary breast cancer correlates with poor prognosis. The aim of this study was to investigate the incidence and the prognostic significance of apoptotic disseminated tumor cells (DTC) in the bone marrow (BM) of breast cancer patients who either underwent primary surgery or primary systemic chemotherapy (PST).

**Methods:**

A total of 383 primary breast cancer patients with viable DTC in the BM were included into this study. Eighty-five patients were initially treated with primary systemic chemotherapy whereas 298 patients underwent surgery first. Detection of apoptotic DTC were performed by immunocytochemistry using the M30 antibody which detects a neo-epitope expressed after caspase cleavage of cytokeratin 18 during early apoptosis. The median follow up was 44 months (range 10–88 months).

**Results:**

Eighty-two of 298 (27%) primary operated patients and 41 of 85 (48%) patients treated with primary systemic systemic therapy had additional apoptotic DTC (M30 positive). In the neoadjuvant group M30-positive patients were less likely to suffer relapse than those without apoptotic DTC (7% vs. 23% of the events, p = 0.049). In contrast, the detection of apoptotic DTC in patients treated by primary surgery was significantly associated with poor overall survival (5% vs. 12% of the events, p = 0.008).

**Conclusions:**

Apoptotic DTC can be detected in breast cancer patients before and after systemic treatment. The presence of apoptotic DTC in patients with PST may be induced by the cytotoxic agents. Thus, both spontaneous and chemotherapy-induced apoptosis may have different prognostic significance.

## Background

30-40% of primary breast cancer patients present with disseminated tumor cells (DTC) at the time of diagnosis and the detection of these cells in the bone marrow has been shown to be a strong independent prognostic factor for disease free and overall survival [1]. Furthermore, DTC are able to survive systemic treatment and their persistence is associated with a poor outcome [2]. However, not all of these patients develop distant metastatic disease during follow-up suggesting that the majority of detected DTC has a short half-life and not the capability to induce tumor growth at secondary sites ("metastatic inefficiency"). Beyond mere detection of DTC, it is therefore important to further characterize these cells with respect to their phenotype and apoptotic status [3].

Apoptosis is a strongly regulated process that occurs in biological organisms leading to destruction of individual cells [4,5]. The role of this programmed cell death in oncogenesis has been intensively investigated in the past two decades. Since the survival of genetically altered cells results in carcinogenesis, an inadequate ratio of apoptosis leads to uncontrolled cell proliferation and thereby tumor growth. This is considered to be a result of mutations in oncogenes which are responsible for the regulation of apoptosis, including BCL-2, C-MYC and P53 [6-8]. Paradoxically, several studies have shown that a high ratio of apoptotic cells in untreated primary breast cancer generally correlates with increased cell proliferation, negative hormonal status, high grading and thus with a poor clinical outcome [8-10]. Whether this phenomenon is restricted to primary tumor or if it takes place in DTC remains to date unclear.

In contrast, chemotherapeutic agents can induce apoptosis of tumor cells leading to disease regression. This process can be explored *in vivo* in patients treated with PST [11]. We have previously reported that PST may induce apoptosis not only in primary tumor, but in DTC as well [3].

The purpose of this study was to investigate the incidence and prognostic significance of apoptotic DTC in two different subgroups of primary breast cancer patients: 1) patients who underwent surgery first and 2) patients treated with PST.

## Methods

A total of 383 primary breast cancer patients treated between 2003 and 2009 at the Department of Obstetrics and Gynecology, University of Tuebingen, Germany were enrolled in this study, which was approved by the local research ethics committee (560/2012R). Inclusion criteria were: non metastatic breast cancer (T1-T3, N0-3, M0) and DTC positive BM status. Patients were subdivided into two groups based on their treatment schedule: (1) patients with surgery followed by adjuvant treatment (n = 298), and (2) patients with PST (n = 85). The clinical characteristics of patients are presented in Tables 1 and 2. The clinical response to PST was assessed by ultrasound, mammography and physical examination and was defined according to the World Health Organization criteria [12]*.* Pathological complete response was considered in patients with absence of invasive tumor in the breast and negative lymph node status. BM aspiration was performed intraoperatively in both groups.

**Table 1:** Clinical data for patients who underwent primary surgery

	**n**	**M30 positive DTC**	**p-value**
	**N = 298**	**(%)**	
Total	298	82 (27)	
Menopausal status			n.s.
Premenopausal	63	14 (22)	
Postmenopausal	235	68 (29)	
Tumor size			n.s.
pT1	204	57 (28)	
pT2-4	94	25 (27)	
Nodal status			n.s.
Negative	214	54 (25)	
Positive	84	28 (33)	
Histology			n.s.
Ductal	226	63 (28)	
Lobular	54	16 (30)	
Others	18	3 (17)	
Grading			n.s.
I/II	268	74 (28)	
III	30	8 (27)	
ER status			n.s.
Negative	45	12 (27)	
Positive	253	70 (28)	
PR status			n.s.
Negative	53	11 (21)	
Positive	245	71 (29)	
HER2 status			n.s.
Negative	232	69 (30)	
Positive	52	12 (23)	

**Table 2:** Clinical data for patients who underwent neoadjuvant therapy

	**n**	**M30 positive DTC**	**p-value**
	**N = 85**	**(%)**	
Total	85	41 (48)	
Menopausal status			n.s.
Premenopausal	48	20 (42)	
Postmenopausal	37	21 (57)	
Tumor size			n.s.
ypT0	19	12 (63)	
ypT1	31	13 (42)	
ypT2-4	35	16 (46)	
Nodal status			0.007
ypN negative	39	25 (64)	
ypN positive	46	16 (35)	
Histology			n.s.
Ductal	66	32 (49)	
Lobular	15	7 (47)	
Others	4	2 (50)	
Grading			n.s.
I/II	67	34 (50)	
III	18	7 (39)	
ER status			n.s.
Negative	28	15 (54)	
Positive	57	26 (46)	
PR status			n.s.
Negative	27	17 (63)	
Positive	58	24 (41)	
HER2 status			n.s.
Negative	66	31 (47)	
Positive	19	10 (53)	

### Collection and analysis of BM

Between 10 and 20 ml of BM were aspirated from the anterior iliac crest and processed within 24 hours. All specimens were obtained after written informed consent from patients. BM samples were separated by density centrifugation over Ficoll (Biochrom, Germany) with a density of 1,077 g/ml. 10^6^ mononuclear cells were spun onto a glass slide using a cytocentrifuge (Hettich, Tuttlingen, Germany). Slides were than fixed in a 4% neutral buffered formalin solution for 10 minutes and were rinsed in phosphate-buffered saline. Automatic immunostaining was performed on the DAKO autostainer using the monoclonal mouse A45–B/B3 antibody (Micromet, Munich, Germany), and the DAKO-APAAP detection kit (DakoCytomation, Glostrup, Denmark) according to the manufacturers’ instructions. For each patient, 2 × 10^6^ cells were analyzed. Slides were automatically scanned using the ACIS™ imaging system (ChromaVision, Medical Systems Inc., San Juan, Capistrano, CA, USA) and evaluated based on the recommendations for standardized tumor cell detection and the criteria of the European ISHAGE Working group [13,14]. The MCF-7 cell line served as a positive control. Leukocytes from healthy volunteers were used as a negative control. All BM specimens were evaluated qualitatively, as positive and negative for DTC.

**Figure 1:**
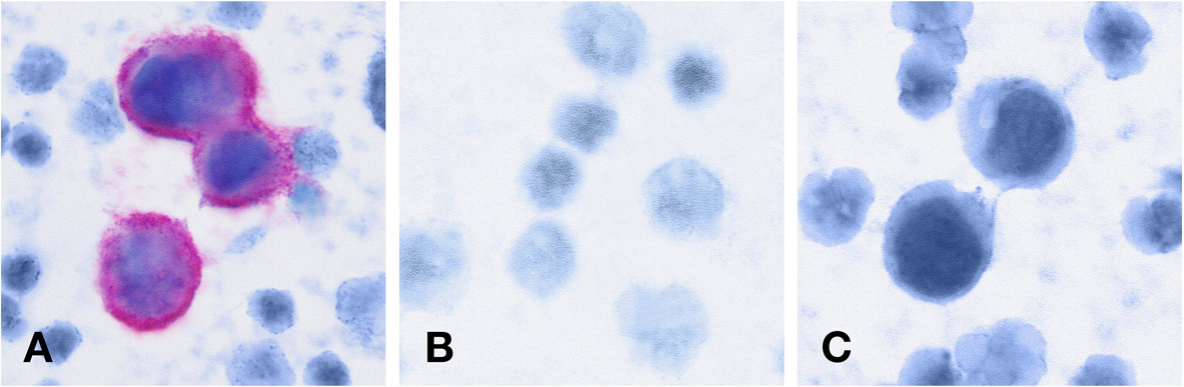
M30 control stainings (A) Cluster of M30 positive apoptotic MCF-7 cells with leukocytes in the background (positive control) (B) Leukocytes from healthy volunteers (negative control) (C) Cluster of M30 negative viable MCF-7 cells with leukocytes in the background (negative control).

**Figure 2:**
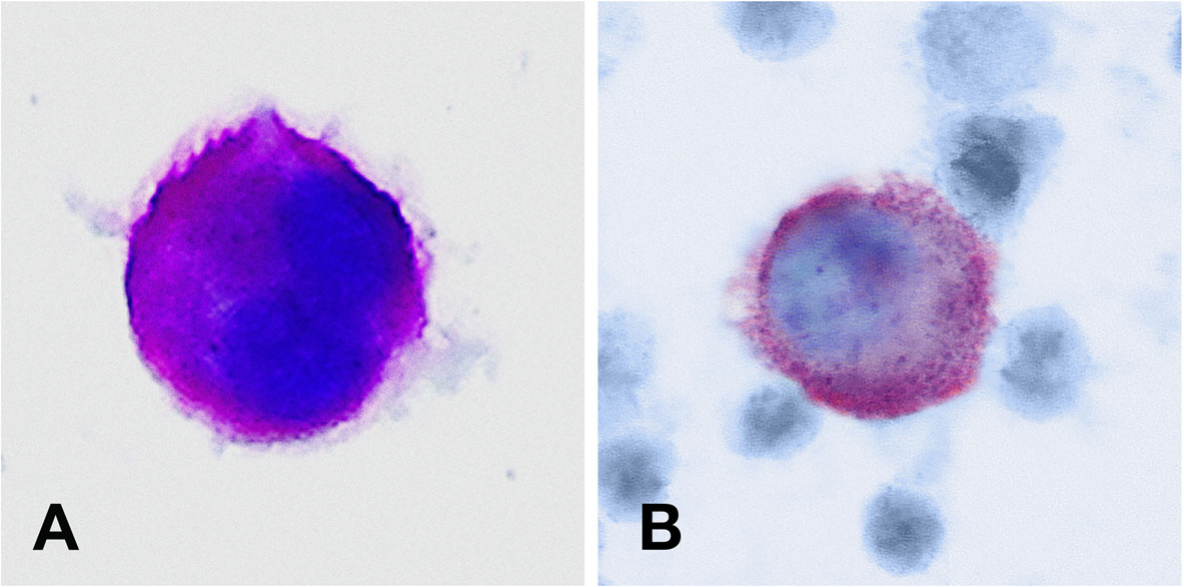
**Pan-cytokeratin and M30 staining of DTC from primary breast cancer patients. (A)** A45–B/B3 positive viable DTC from a primary breast cancer patient **(B)** M30 positive apoptotic DTC from a primary breast cancer patient. The typical morphology of a tumor cell can be recognized (positive cytokeratin-staining, large nucleus, high nuclear to cytoplasmic ratio, nucleus partially covered by cytokeratin staining, nucleus granular).

383 primary breast cancer patients with A45-B/B3 positive DTC in BM were included into this study. In order to evaluate the apoptotic status of DTC in this group, additional BM slides were stained using the M30 antibody (Roche Applied Science, Mannheim, Germany) and analyzed by use of the APAAP kit detection method as described above. The M30 antibody reacts with a neo-epitope expressed only after caspase cleavage of cytokeratin 18 during early apoptosis [15]. M30 antibody does not bind intact, full-length cytokeratin 18 in viable or necrotic cells and can, therefore, be used specifically to recognize apoptotic cells [16]. Identification of apoptotic DTC was based on positive M30 staining and cytomorphological criteria as described elsewhere [17-19]. MCF-7 cells treated with sodium azide served as a positive control; untreated MCF-7 cell line and leukocytes from healthy volunteers were used as a negative control. Figures 1 and 2 show M30 and pan-cytokeratin staining.

### Statistical analysis

The chi-squared test was used to evaluate the association between apoptotic DTC and clinicopathological factors. Statistical analysis was performed by SPSS (version 19). Values of *p* < 0.05 were considered statistically significant. Survival intervals were measured from the time of BM biopsy until death or the first diagnosis of relapse. Relapse was defined as either local recurrence or distant metastasis. Survival was calculated using Kaplan-Meier method and compared by the log-rank test.

## Results

### Patients characteristics

A total of 383 DTC positive breast cancer patients were included in this study. Two-hundred ninety-eight patients underwent breast surgery first. 204 out of 298 patients (68%) had T1 tumors and 214 (71%) were node negative. The most common histological tumor type was invasive ductal carcinoma. Estrogen and progesterone receptor status were positive in 85% and 82% of these patients, respectively. Fifty-two of 284 (18%) patients had HER2 positive tumors. Clinical data of this group are summarized in Table 1. Eighty-five patients were treated with PST. The majority of these patients was premenopausal (48 of 85 cases). 22% achieved pathological complete response after chemotherapy while 53% responded partially. Stable disease was observed in 19% of patients whereas 6% of patients (5 cases) developed progressive disease (Table 3). Clinical data of patients treated with PST are summarized in Table 2.

**Table 3:** Apoptotic DTC and clinical response to neoadjuvant chemotherapy

	**N (%)**	**M30 positive DTC**	**%**
Total	85 (100)	41	48
Complete remission	19 (22)	12	63
Partial remission	45 (53)	24	53
Stable disease	16 (19)	5	31
Progressive disease	5 (6)	0	0

### Presence of apoptotic DTC in patients treated with primary surgery

In eighty-two of 298 (27%) patients with pan-cytokeratin positive DTC in BM who underwent primary surgery additional apoptotic DTC could be detected. No correlation could be found between positive M30 status and any established prognostic factors, including tumor size, lymph node status, hormone receptor status or grading.

### Presence of apoptotic DTC in patients treated with PST

Forty-one of 85 (48%) patients had additional M30 positive DTC after completion of PST. Patients with apoptotic DTC were less likely to have nodal metastasis (35% vs. 64%; p = 0.007). No significant correlation could be observed between the positive M30 status of DTC and other clinicopathological factors. The presence of apoptotic DTC was associated with response to PST. M30 positive cells were found in 63% of patients with complete remission, 53% with partial remission and 31% with stable disease, respectively. None of the patients with progressive disease had M30 positive DTC (p = 0.034; Table 3).

### Survival analysis

The median follow-up was 44 months (range: 10–88 months). 32 of 383 patients were diagnosed with relapse (either local recurrence or distant metastasis) and 28 died during follow-up. Clinical outcome data are summarized in Table 4.

**Table 4:** Survival analysis of patients depending on M30 status of DTC

	**Patients with primary surgery**	**Patients with NST**
N	298	85
Deaths	21	7
M30 positive	10/82 (12%)	4/41 (10%)
M30 negative	11/216 (5%)	3/44 (7%)
P	0.032	n.s.
Overall survival*		
(M30 positive vs M30 negative)	0.008	n.s.
Relapses†	19	13
M30 positive	7/82 (9%)	3/41 (7%)
M30 negative	12/216 (6%)	10/44 (23%)
P	n.s.	0.049
Relapse free survival*	n.s.	0.128
(M30 positive vs M30 negative)		

*Calculated by log-rank test. † Including local recurrence and distant metastases.

### Survival analysis of neoadjuvant patients

Thirteen of 85 neoadjuvant patients (15%) presented with relapse during follow-up. Patients with additional M30 positive DTC were less likely to suffer from relapse than patients with only non-apoptotic DTC (7% vs. 23%; p = 0.049). However, the association between disease-free interval and M30 status of DTC assessed by Kaplan-Meier analysis did not reach statistical significance (p = 0.128; 70 months, 95% CI: 63–78 months vs. 81 months, 95% CI: 74–87 months). Seven of 85 neoadjuvant patients (8%) died during follow-up. No correlation could be found between M30 status of DTC and overall survival in this group of patients.

### Survival analysis of patients treated with primary surgery

**Figure 3:**
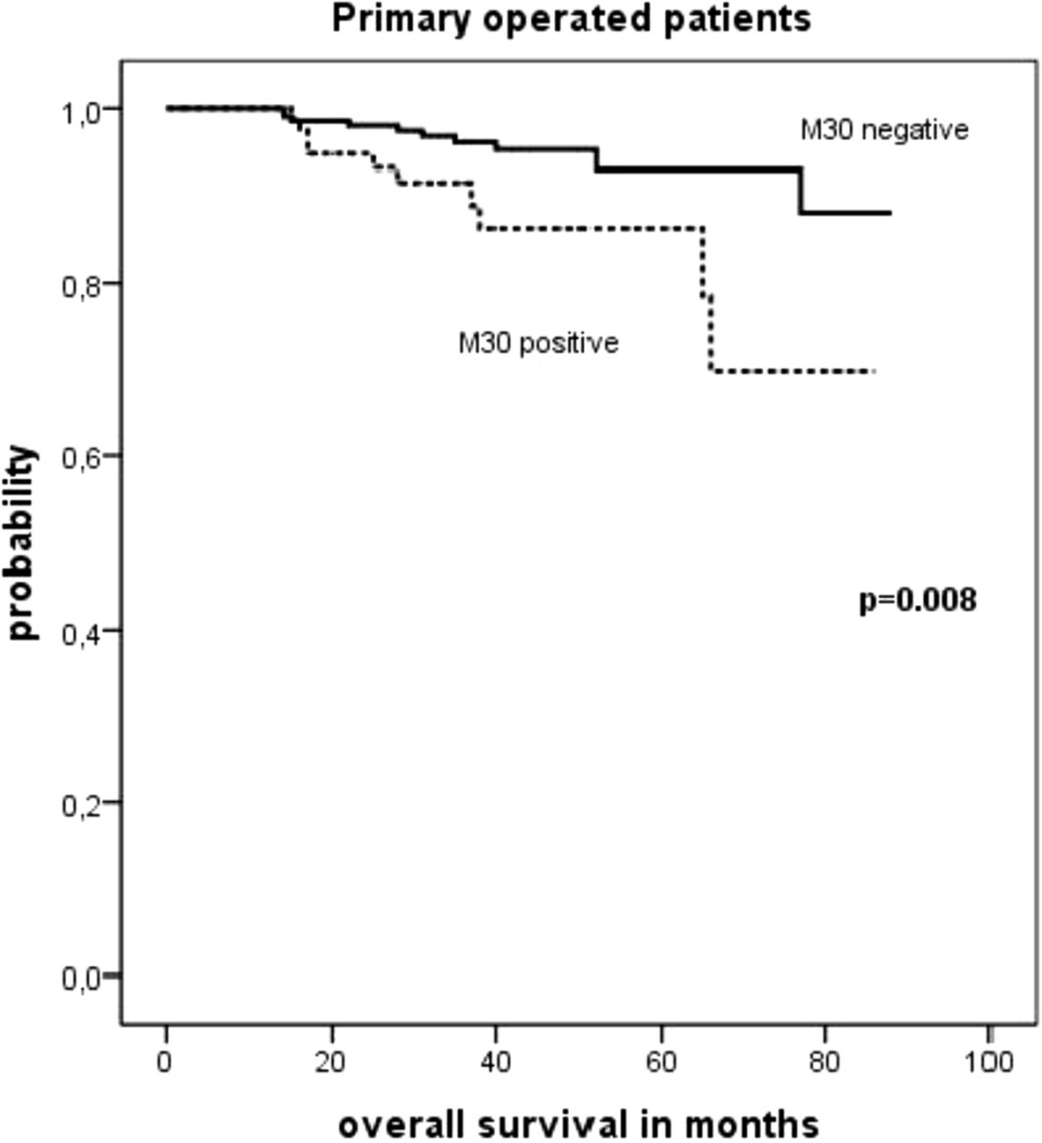
Kaplan-Meier survival analysis depending on M30 status of DTC in bone marrow of previously untreated patients (overall survival).

Twenty-one out of 298 (7%) patients in this group died during follow-up. The overall survival was significantly shorter among patients with M30 positive DTC as compared to M30 negative patients (75 months, 95% CI: 68–81 months vs. 84 months, 95% CI: 81–86 months; p = 0.008). However, there was no association between disease free survival and M30 status (83 months in M30 positive patients, 95% CI: 80–85 months vs. 78 months in M30 negative patients, 95% CI: 73–84 months; p > 0.05). Figure 3 shows the Kaplan-Meier analysis of overall survival in patients who underwent primary surgery.

## Discussion

The presence of DTC in BM of patients with primary breast cancer is an independent prognostic factor associated with poor clinical outcome [1]. Although this phenomenon can be seen in 30-40% of breast cancer patients, only a minority of DTC positive patients will develop distant metastasis in course of disease ("metastatic inefficiency").

The aim of the present study was to evaluate the incidence and prognostic relevance of apoptotic DTC in breast cancer patients. We focused on two subsets of patients: 1) untreated patients whose bone marrow status was assessed at the time of primary surgery, and 2) pre-treated patients after completion of neoadjuvant cytotoxic therapy.

### Clinical relevance of apoptotic DTC in untreated patients at time of primary surgery

M30 antigen is an early apoptotic marker of epithelial cells, detectable after caspase cleavage of cytokeratin 18. This antibody was used in our study to assess the apoptotic status of DTC in 298 patients who received no treatment prior to surgery. Patients with apoptotic DTC had a significantly shorter overall survival compared to patients with only non-apoptotic DTC (75 months vs. 84 months, p < 0.008). This seems not conclusive, since apoptotic DTC are generally assumed to result from a phenomenon described as "metastatic inefficiency". According to this conceptual model, only a small percentage of tumor cells are able to survive and persist at secondary homing sites. Large numbers of cancer cells are shed from the primary site into the systemic circulation; only a small subset will give rise to overt metastases. To successfully reach and colonize a secondary site, a tumor cell must complete a series of steps (metastatic cascade): migration from the primary tumor, intravasation into the blood stream, survival of the vigorous passage in blood, extravasation, and development of micrometastases in distant organs [20]. Failure in any one of these steps leads to elimination of tumor cells; 99,9% of shed cells are thought to perish during the process, while only a minor subpopulation attains metastatic capacity [21]. The key regulatory points that contribute to metastatic inefficiency remain unclear; initiation of apoptosis has been assumed to be a major component of this mechanism. Yet, in our study apoptotic status of DTC in bone marrow in untreated patients resulted in significantly worse survival.

In the past decades, major research efforts have been conducted to study the role of apoptosis in the primary tumor [10,22]. The association between apoptosis rates and cell proliferation is well established; increased apoptosis reflects a high cell turnover in the tumor. In breast cancer, high levels of apoptosis correlate with enhanced cellular proliferation and biological markers of increased malignancy, such as negative hormone receptor status, high histological grade, HER2 overexpression, positive lymph nodes, tumor aneuploidy and a decreased expression of bcl-2 protein [10,23,24]. Furthermore, high apoptotic counts are associated with shortened disease-free and overall survival [9,10,24]. Similar observations were made in other solid tumors, including prostate and bladder cancer [25,26]. These data seem to disprove the concept that the elimination of apoptosis in tumor cells is a necessary condition for autonomous uncontrolled cancer growth. On the contrary, an enhanced rate of spontaneous apoptosis in the primary tumor is an indicator of high proliferation and negative prognostic markers.

### Clinical relevance of therapy-induced apoptosis in DTC

Over the last two decades, neoadjuvant systemic therapy (NST) has become the standard treatment strategy for locally advanced breast cancer, conducted primarily to enhance the possibility of breast-conserving surgery [27]. Beyond this indication NST offers the additional possibility to test in vivo the chemosensitivity of the primary tumor [28]. The pathological response to NST is associated with favorable clinical outcome and is considered by some as a surrogate marker for complete eradication of micrometastatic disease. However, up to 25% of patients who achieve complete pathological remission will suffer relapse within five years of diagnosis, suggesting subclinical persistence of isolated tumor cells beyond systemic treatment.

We reported previously a high incidence of persistent DTC after NST [3]. Since apoptosis is the main mechanism of chemotherapy-induced disease regression [29], we aimed to assess the apoptotic status of DTC in patients who received neoadjuvant therapy. In 41 out of 85 (48%) DTC positive patients additional apoptotic tumor cells were detected after therapy. Compared to patients with only viable DTC, patients with apoptotic DTC responded better to therapy, reaching complete or partial remission in 88% (vs. 64% in the M30-negative group, p = 0.034). Moreover, additional apoptotic cells in BM were significantly associated with negative nodal status after completion of neoadjuvant treatment but not with any other clinicopathological factor. Pathological response of the primary tumor and lymph nodes metastases was therefore reflected by changes in tumor cells in secondary sites, such as bone marrow. Further, patients with positive M30-status were less likely to suffer from a relapse (p = 0.049). This is in accordance with clinical studies: patients who achieve complete pathological response to neoadjuvant treatment regime perform favorably with regard to overall and disease-free survival [30]. In addition, patients converted to node negative disease after neoadjuvant treatment have high survival rates, despite residual tumor in the breast [31]. These data indicate that in these patients apoptotic tumor cells are associated with therapy response.

## Conclusions

Although the observations made in primary tumors cannot be directly extrapolated to DTC in secondary sites, our data suggest that high level of spontaneous apoptosis in minimal residual disease (MRD) is an indicator of poor prognosis. Hypothetically, if the presence of apoptotic DTC reflects an active status of MRD; accordingly, dormant (inactive) tumor cells would appear as non-apoptotic. In contrast, therapy-induced apoptosis of DTC was correlated to pathological response of the tumor and may be regarded as a favorable event. Our data demonstrate for the first time that the biological significance of apoptotic status of DTC is contingent on whether the apoptosis occurs spontaneously or was induced by treatment.

## Abbreviations

ACIS: Automated cellular imaging system; APAAP: Alkaline phosphatase-antialkaline phosphatase; BM: Bone marrow; CK: Cytokeratin; CTC: Circulating tumor cell; DTC: Disseminated tumor cell; HE: Hematoxylin-eosin; MRD: Minimal residual disease; NST: Neoadjuvant systemic therapy; n.s.: Not significant.

## Competing interests

The authors declare that they have no competing interest.

## Authors’ contribution

NK designed the study, performed the statistical analysis and drafted the manuscript. AH made substantial contributions to interpretation of data, performed the survival analysis and helped to draft the manuscript. MB and FMS made substantial contributions to interpretation of data and helped to draft the manuscript. AS made substantial contributions to interpretation of data. MW, CR, JH and MH participated in data collection and study coordination. TF designed the study and critically revised the manuscript for important intellectual content. All authors read and approved the final manuscript.

## Pre-publication history

The pre-publication history for this paper can be accessed here:

http://www.biomedcentral.com/1471-2407/14/394/prepub
